# Control of the conformations of ion Coulomb crystals in a Penning trap

**DOI:** 10.1038/ncomms3571

**Published:** 2013-10-07

**Authors:** Sandeep Mavadia, Joseph F. Goodwin, Graham Stutter, Shailen Bharadia, Daniel R. Crick, Daniel M. Segal, Richard C. Thompson

**Affiliations:** 1 QOLS Group, Department of Physics, Imperial College London, South Kensington Campus, London SW7 2AZ, UK

## Abstract

Laser-cooled atomic ions form ordered structures in radiofrequency ion traps and in Penning traps. Here we demonstrate in a Penning trap the creation and manipulation of a wide variety of ion Coulomb crystals formed from small numbers of ions. The configuration can be changed from a linear string, through intermediate geometries, to a planar structure. The transition from a linear string to a zigzag geometry is observed for the first time in a Penning trap. The conformations of the crystals are set by the applied trap potential and the laser parameters, and agree with simulations. These simulations indicate that the rotation frequency of a small crystal is mainly determined by the laser parameters, independent of the number of ions and the axial confinement strength. This system has potential applications for quantum simulation, quantum information processing and tests of fundamental physics models from quantum field theory to cosmology.

## Abstract (web summary)

Ion traps form the core of many studies of fundamental physics and are a possible route to quantum computation. Using a Penning trap, Mavadia *et al.* demonstrate the formation and control of different configurations of ion Coulomb crystals, which may be useful for future ion-based applications.

## Graphical abstract

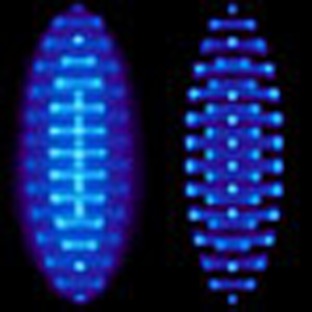

Ion Coulomb crystals (ICCs) are formed whenever trapped atomic ions reach temperatures low enough that their thermal energy is much less than the Coulomb repulsion between the ions. This is usually achieved using Doppler laser cooling, which gives final temperatures of the order of 1 mK. It is convenient to introduce the Coulomb coupling constant Γ=*e*^2^/(4*πε*_0_*ak*_B_*T*), where *a* is the Wigner–Seitz radius defined through 4*πa*^3^*n*_0_/3=1, *n*_0_ is the number density of ions and *k*_B_ is the Boltzmann constant. Monte Carlo simulations show that large, three-dimensional (3D) clouds of ions form solid-phase crystals at Γ≈172–178 (see ref. 1 and references therein) and at Γ≈140 for two-dimensional (2D) systems^2^. In these crystals, the inter-ion distance is typically of the order of 10 μm, set by the strength of the applied trapping fields. The density is therefore many orders of magnitude less than in usual condensed matter crystals and the dynamics are completely determined by electromagnetic forces, independent of the atomic structure of the ions.

A great deal of work with ICCs has been performed in linear radiofrequency (RF) traps, where the confinement perpendicular to the trap axis is provided by an oscillating potential applied to the trap electrodes. The trapping force along the axis of the trap is due to a static potential typically produced by applying a DC voltage to additional electrodes at the ends of the trap (‘endcaps’). If this axial trapping force is set to be much weaker than that in the radial direction and a small number of ions is held in the trap, they will form a linear string along the trap axis. If the oscillation frequency along the trap axis is increased by raising the voltage applied to the endcaps, there comes a point at which the string adopts a zigzag structure. For even tighter axial confinement, the ions can form a 3D solid crystal. Such crystals take a variety of shapes dependent on the number of ions and the trapping parameters^3^.

In recent years, linear strings of ions in RF traps have been used for the deterministic generation of entanglement between massive particles, leading to applications in quantum information processing (see, for example, ref. 4). Beyond this, other forms of ICCs in RF traps have been used for a variety of experiments, including cavity quantum electrodynamics (QED)^5^ and the measurement of reaction rates of ions with molecules^6^. Many further proposals have been made for the use of ICCs in direct quantum simulation of a wide variety of physical systems and processes, from quantum spin systems^7^ to Hawking radiation^8^. A few such ideas have been implemented experimentally, including the simulation of a quantum magnet^9^ and simulations of quantum walks^10^. Of particular interest to the work presented here is a body of theoretical work that considers quantum mechanical aspects of linear-to-zigzag transitions in ion chains^11^. Recently, there has been a great deal of interest in the formation of defects in zigzag chains of ions in RF traps^12^ as a means of studying the inhomogeneous Kibble–Zurek mechanism^13^,^14^,^15^.

One consequence of the radial trapping mechanism in the linear RF trap is that any trapped particles that do not lie exactly on the trap axis execute a small-amplitude driven motion at the applied trap frequency (the ‘micromotion’). The micromotion amplitude increases with distance from the axis and, therefore, leads to an oscillation in the relative positions of the ions in the crystal, which is absent in the Penning trap (see below).

Apart from the two-ion ICC reported in ref. 16, until now ICCs studied in Penning traps have been relatively large (containing between hundreds and hundreds of thousands of ions)^17^,^18^. The Penning trap has a number of advantages for some kinds of experiments. It is the ideal tool for precision measurements on ions that require a large magnetic field, for example, the measurement of the bound-state electron *g*-factor^19^ or the measurement of dipole moments of molecular ions from shifts to the cyclotron frequency due to their polarizability^20^. However, it may also have some advantages for certain proposed experiments with ICCs where the interest lies in the sensitive dynamics of the transitions between different crystal conformations that occur when the trapping potential is varied^11^,^21^.

The main advantage of the Penning trap for such studies lies in the fact that it employs purely static electric and magnetic fields. The lack of RF fields means that ICCs can be studied in the absence of driven oscillations, which could obscure the dynamics of interest, in particular when 2D or 3D structures are required. Furthermore, the stiffness of the trap is set by the strength of the magnetic field and by a modest DC electric potential. This removes the usual requirement in RF traps for small electrode-ion distances that can lead to heating due to surface effects^22^, which, despite recent progress^23^, remain relatively poorly understood. The limiting factor to the stiffness of the trap is the magnetic field strength available. For magnetic fields in the region 1–5 T, the secular trap frequencies are still somewhat lower than those achievable using miniature RF traps. Despite this limitation, ICCs in Penning traps have potential applications for quantum simulation, quantum information processing and tests of fundamental physics models from quantum field theory to cosmology^17^,^21^.

Here we demonstrate the creation of ICCs in a Penning trap, containing up to 30 laser-cooled ions. We control the conformations of the crystals by adjusting the trap parameters. From comparisons of images of the ICC with the results of simulations, we are able to determine the frequency at which the ICC rotates. This in turn depends mainly on the laser cooling parameters for the small crystals considered here. In contrast to earlier studies, there are no oscillating fields present in this system.

## Results

### Motivation and Penning trap techniques

Optical sideband cooling of a single ion in an RF trap down to the ground state of its motion in three dimensions was first performed nearly 20 years ago^24^. This led to an explosion of interest in trapped ions as systems that are ideal for experiments in quantum information processing and simulation. As yet, optical sideband cooling has not been implemented in a Penning trap. The experiments we report here pave the way to the implementation of this important technique for single ions and small ICCs in Penning traps. This in turn should enable a variety of very interesting potential applications to be realized. To give one example, three ions in a Penning trap will naturally form a radial crystal with a triangular symmetry, which lends itself to the simulation of frustration in condensed matter systems^25^.

The Penning trap used in this work consists of a stacked set of ring-shaped electrodes, which produce a DC quadrupole electric field to confine the ions in the axial direction (see Methods). Confinement in the radial plane is provided by a uniform magnetic field (*B*=1.85 T) produced by a superconducting solenoid magnet. This trapping configuration produces no micromotion; however, one consequence of the presence of a strong magnetic field is that the whole crystal rotates in the trap. The density of ions varies with this rotation frequency Ω_rot_/2*π*, taking a maximum value when the rotation is at exactly half the pure cyclotron frequency^26^. The cyclotron frequency for ions of charge to mass ratio *e*/*m* is given by Ω/2*π*=*eB*/2*πm*. The maximum density can be achieved either by the application of an additional rotating potential (see below), which phase locks the crystal rotation, and can be used to spin the crystal up to half the cyclotron frequency (referred to as Brillouin flow)^27^,^28^, or by applying a torque to the ions using a laser beam^29^.

The radial motion of a single ion in a Penning trap consists of two modes: modified cyclotron and magnetron. The radius of an ion’s magnetron orbit around the trap axis increases as this mode loses energy. During laser cooling, the size of the unstable magnetron orbit can be kept small by ensuring that the laser beam has an intensity profile such that the intensity is greater where the magnetron motion is in the same direction as the photon momentum^30^. In practice, this means that the radial laser beam needs to be displaced slightly from the axis of the trap. For an ion crystal the situation is similar, although all the ions now rotate around the magnetic field vector. The laser exerts a torque on the ions and the crystal rotation frequency finds a steady-state equilibrium value where the integrated value of the torque over a complete orbit is zero. Note that torques on the ion cloud can also arise from imperfections in the trap construction leading to higher-order terms in the electrostatic potential, but they are expected to be small here because of the small size of the crystal.

ICC experiments in Penning traps have mainly focused on very large crystals of ions, taking advantage of the absence of micromotion (which can cause increased heating with larger ICCs in RF traps). The structures of the crystals have been investigated by direct imaging and also by Bragg scattering^18^. In that work, the bulk crystal was found to show a body-centred cubic structure. It is also possible to prepare the crystal as a 2D planar structure by adjusting the trap parameters appropriately^31^. In this case, the larger crystals generally form a triangular lattice. This system has been used for spectroscopy of the transverse modes of oscillation of the crystal (which is akin to a drumhead^31^). A simulation of a 2D Ising interaction has also been carried out with such 2D crystals in a Penning trap^17^.

For experiments with large crystals or clouds of ions in a Penning trap, it has been found to be useful to apply additional fields to enable precise control of the rotation of the crystal^17^ or to maximize the number density *n*_0_ (ref. 27), which, for a solid crystal, is linked to the angular rotation frequency Ω_rot_ through the relation^26^


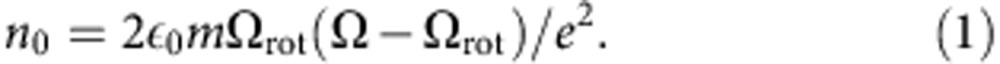


In the ‘Rotating Wall’ technique, a radial dipole or quadrupole field rotating about the trap axis is used for this purpose^27^,^28^. As stated above, the maximum density of ions is obtained when the applied rotating wall angular frequency is Ω/2. An alternative technique that has been used to improve the laser cooling of single ions and small clouds of ions is ‘axialization’ where an oscillating radial quadrupole field is applied at angular frequency Ω (ref. 16). As this field can be decomposed into two counter-rotating quadrupole fields rotating at ±Ω/2, the technique is closely related to the Rotating Wall technique. These additional fields necessarily distort the crystal to control its motion. There is clearly an advantage to avoiding the use of these additional fields if possible, as oscillating potentials applied to crystals can lead to additional motion if the RF null is not aligned with the centre of the crystal. No RF fields additional to the static Penning trap fields were used in the experiments described here.

### Images of ICCs

**Figure 1:**
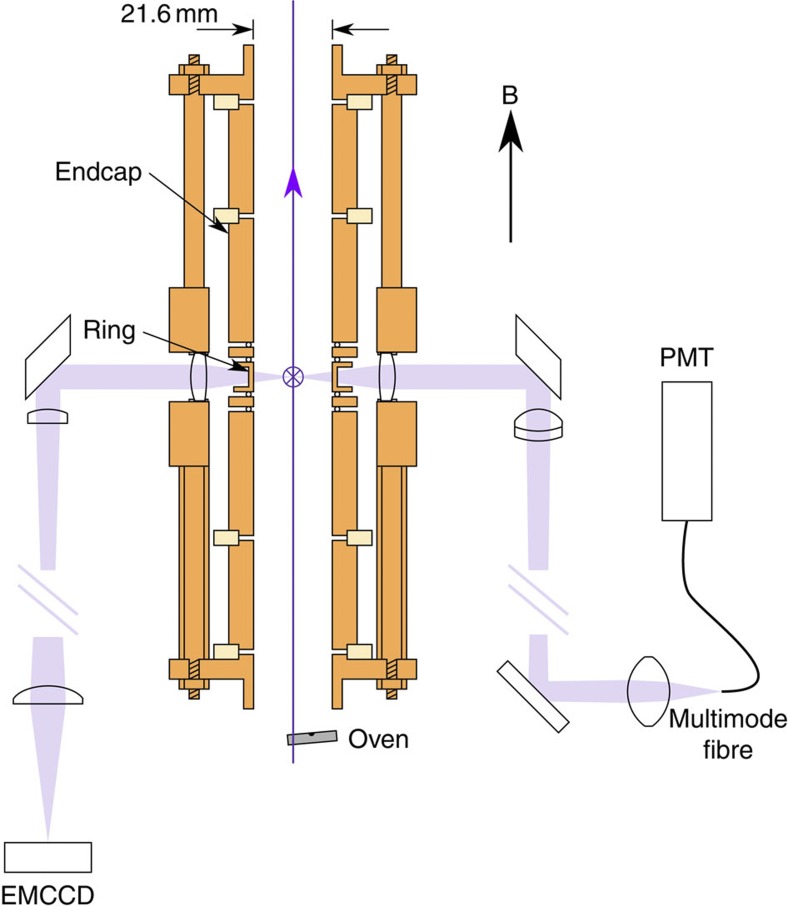
Trap schematic. Penning trap layout showing cooling laser beams and fluorescence detection optics. A cross-section of the electrodes is shown. The vertical purple line is the axial cooling beam. The radial cooling beam passes through holes in the ring electrode, points into the page and is marked with a purple cross. The path taken by the atomic fluorescence is indicated by light purple shading. The internal diameter of the trap is 21.6 mm. The trap, vacuum enclosure (not shown) and beam-steering optics fit inside the 105-mm vertical bore of the superconducting magnet. EMCCD, electron multiplying charge-coupled device; PMT, photomultiplier tube.

We load small numbers of ^40^Ca^+^ ions into our trap. The ions have a cyclotron frequency Ω/2*π*=705 kHz. For details of the lasers used for cooling, see Methods. The apparatus has been described before^27^, but here we report the addition of an axial laser cooling beam to our setup (see Fig. 1), which has proven to be an essential requirement for the production of the ion strings and 3D ICCs reported here (see Methods). Axial cooling has also been shown to be important in previous Penning trap experiments with larger ICCs (for example, refs 26, 29)

**Figure 2:**
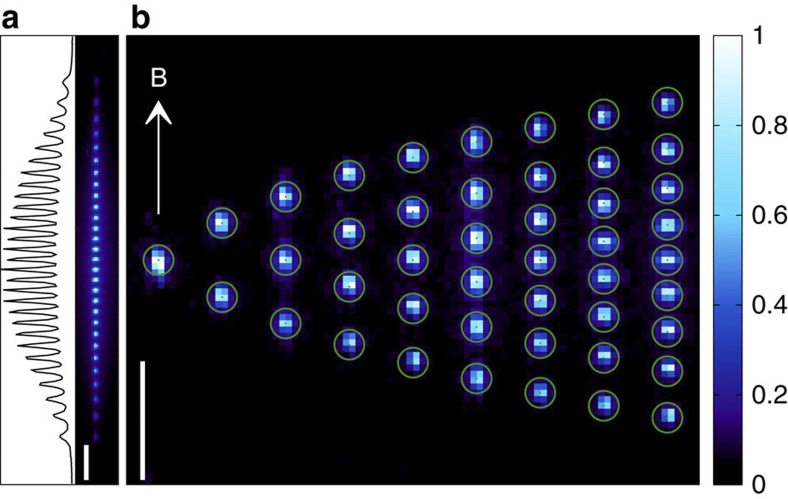
Linear ion chains. (**a**) Image and intensity profile of a chain of 29 ions. The ions at the end of the chain are less bright than the central ions because of imperfections of the imaging system and because they are not as well overlapped with the radial laser beam, which has a diameter of ~100 μm. (**b**) Collage of linear crystals of one to nine ions. The applied voltage was kept constant for all of these experimental images, corresponding to a normalized axial trapping frequency 
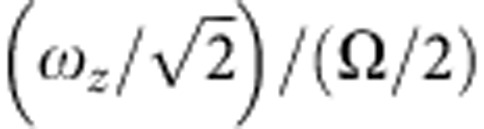
=0.158. Each image is an accumulation of twenty 1-s exposures and the maximum intensity is normalized to unity for each image. Each pixel is equivalent to 2.65±0.15 μm in the centre of the trap. The circles around the ions are the calculated positions, from ref. 32, and not from a fit to the data. The bars at the bottom of the images represent a length of 50 μm.

To load the trap, neutral calcium atoms from a thermal beam are ionized by pulses from a frequency-doubled Nd:YAG laser. We are able to deterministically prepare crystals containing countable numbers of ions from roughly 30 down to 1. For larger ICCs with more than 100 ions, the number of ions can be inferred through comparisons with simulations (see Methods). At low applied voltages, the ions always form an axial string. We have been able, for the first time, to stably trap and image in a Penning trap ion strings up to 0.46 mm in length (29 ions). Figure 2 shows images of linear ICCs. The circles are calculated positions^32^ and they agree well with the actual positions of the ions within the uncertainty set by the finite optical resolution, implying that the trap potential in the *z* direction is well approximated by a harmonic potential. Nine ions were initially loaded and the axial potential set low so that a string would form. Ions were then ejected in a controlled manner so as to create strings of the required length. Figure 2 also shows the longest linear chain we have trapped (29 ions), although this is not a fundamental limit.

**Figure 3:**
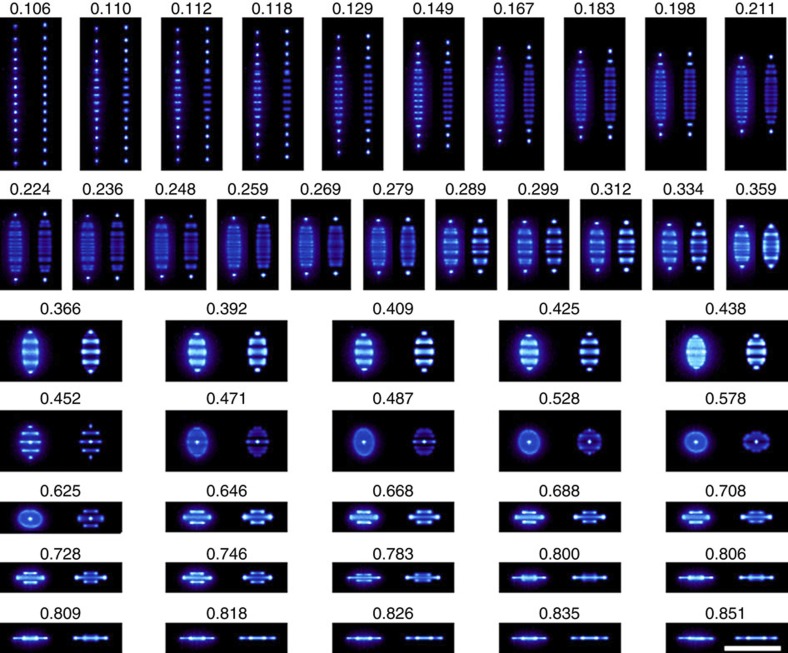
Fifteen-ion crystals for different axial potentials. Experimentally obtained images of 15-ion crystals (left of each panel) compared with computer simulations (right of each panel). By increasing the axial confinement, a linear string is transformed into a zigzag structure, then a 3D crystal and finally a planar structure. Each image is labelled with the value of the normalized axial trapping frequency, 
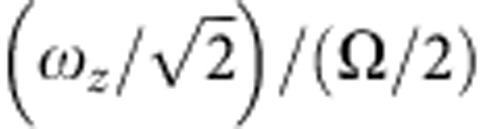
 (the trap becomes unstable when this quantity is equal to unity). There is a 100-μm scale bar in the bottom right-hand pane, which applies to all the images.

A set of experimentally obtained images of ICCs in our trap is shown in Fig. 3. For this sequence, 15 ions were loaded into the trap and the trapping voltage, which sets the axial frequency *ω*_*z*_ (see Methods for definition), was varied. The laser parameters were unchanged throughout. As the axial confinement is increased, at a certain applied voltage the ion string begins to adopt a visibly zigzag-type structure, as expected from simulations and observations of other systems. We have carried out our own simulations to determine the equilibrium configuration of an ensemble of ions in the trap at low temperatures (see Methods). In Fig. 3, each experimentally obtained image is shown alongside a simulated image. As the now zigzag structure is imaged from the side, and the magnetic field causes it to rotate with a period short compared with the exposure time of the electron multiplying charge-coupled device camera, the image of each ion spreads into a line. With still tighter axial confinement, the ions form 3D structures and, finally, for the highest values of *ω*_*z*_ the crystal comprises a single layer in the radial plane.

The ICCs shown in Figs 2 and 3 are remarkably stable. With the cooling lasers on, they remain unchanged indefinitely. If the laser beams are blocked for a short period (for example, 1 s), the ICCs reappear unchanged essentially immediately once the laser radiation has been restored. For large ICCs, the aspect ratio of the crystal has been shown to provide information about the rotation frequency^26^,^28^,^33^. This is because the radial confinement depends on the rotation frequency; thus, its value can be inferred from the shape of the cloud. The simulations we perform support this general conclusion but give more accurate estimates of the rotation frequency than one would obtain from using fluid theory for small ICCs. There is a limited number of distinct conformations for small ICCs. For a given conformation, the accuracy to which the rotational frequency can be determined is limited by the imaging resolution.

### Rotation frequency and ion number density

Considerable insight can be gained by considering the dynamics in a frame that is rotating at half of the cyclotron frequency (that is, at Ω/2)^34^. In this rotating frame, the Lorentz force due to the magnetic field is exactly cancelled by the fictitious force due to the rotation. This also modifies the radial potential such that the effective radial potential in this frame gives an harmonic oscillation frequency of 
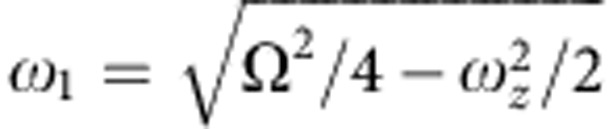
. In the rotating frame, the ions therefore behave as if they are in a 3D potential well with no magnetic field. Note that unlike in RF traps, no pseudopotential approximation is required in this treatment and the trap potential automatically has exact cylindrical symmetry. If the ion crystal has the highest possible density (Brillouin flow^27^), it will be stationary in this frame and it will be rotating in the laboratory frame at Ω/2. If, however, the crystal is not stationary in the rotating frame, its observed angular rotation frequency (Ω_rot_) will not equal Ω/2 and the density will be lower. In the rotating frame, this drop in density can simply be seen as the consequence of an outward (centrifugal) force on each ion arising from its orbit of the trap centre. This approach also leads to the above expression (equation 1) linking *n*_0_ to Ω_rot_.

If 
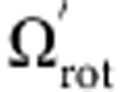
 is the angular frequency at which the crystal is rotating in the rotating frame, then we can relate this to the observed angular rotation frequency in the laboratory frame Ω_rot_ through


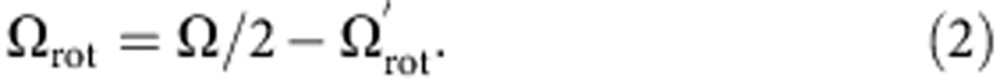


This assumes that Ω_rot_<Ω/2, which is true for all the experiments reported here. The effective harmonic trap frequency in the crystal frame, that is, the frame in which the crystal is stationary, is given by


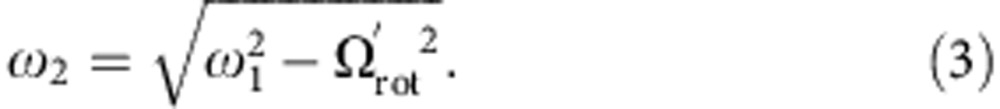


**Figure 4:**
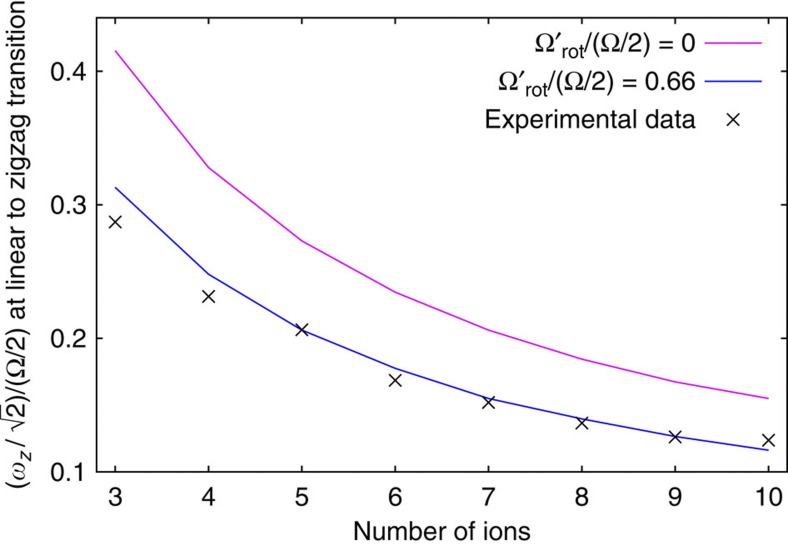
Transition from linear to zigzag structure. Plot of the measured values of 
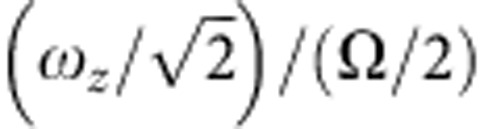
 at the onset of a zigzag structure for different lengths of chain. The solid lines follow theoretical predictions calculated for different values of the rotation frequency of the crystal. The best fit suggests that these crystals were rotating at ~0.34 × Ω/2 in the laboratory frame or 0.66 × Ω/2 in the rotating frame.

Our simulations take into account this effective reduction in radial confinement arising from any rotation of the crystal in the frame that is itself rotating at Ω/2 with respect to the laboratory. We find that there is good agreement between the simulated and the observed configurations when we take this rotation into account. For example, we find in our simulations that the first onset of the zigzag shape of the ion string comes at a lower value of applied voltage than would be expected, based on the effective potential in the rotating frame. Figure 4 is a plot of the measured values of 
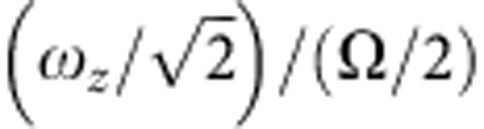
 at which the linear-to-zigzag transition occurs, for strings of different numbers of ions. The laser parameters remained approximately the same for all the data presented in Fig. 4. It is clear from the plot that these zigzag crystals are neither stationary in the laboratory frame nor in the rotating frame. A theoretical analysis of the effect of the laser cooling beam on the crystal, based on the ideas presented in ref. 35, shows that the actual value of the rotation frequency in the laboratory frame is dependent on the laser-cooling parameters and can be estimated in our trap to be anywhere within the range 35–280 kHz (that is, Ω_rot_=(0.1–0.8) × Ω/2), depending on the saturation, offset and detuning of the laser beams. This analysis only applies in the limit that the radius of the crystal is less than the radial laser beam waist (~30 μm).

**Figure 5:**
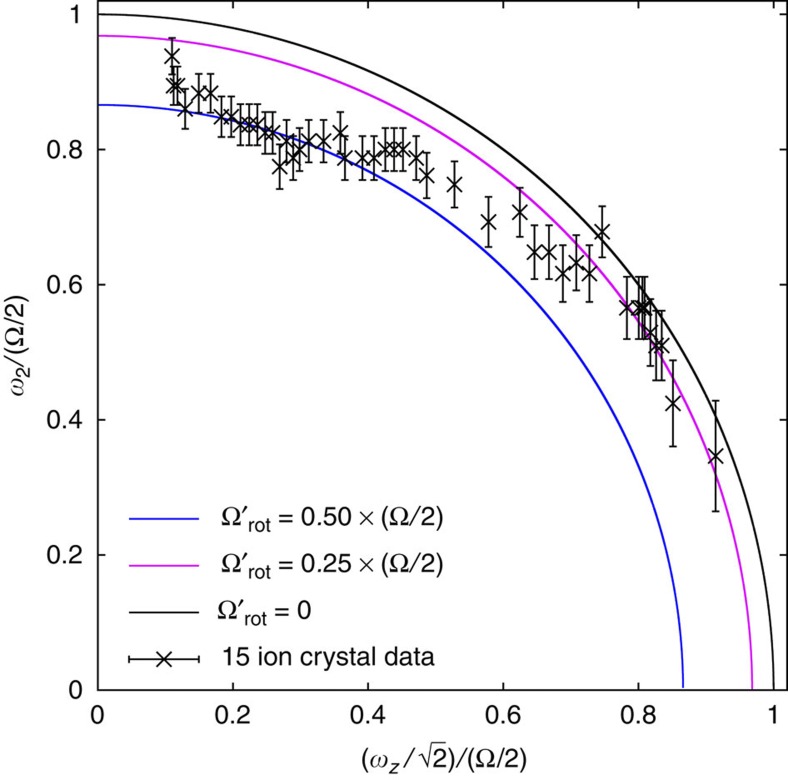
Determination of the radial trapping frequency. Plot of the radial trapping frequency in the crystal frame against the axial trapping frequency. The data are derived by comparison of experimental images with simulations. The error bars are derived from how accurately the radial trap frequency can be determined by comparison with the computer simulations. Lines of constant 
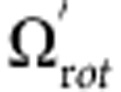
 are shown for comparison. For the chosen scaling, these lines are circular arcs.

Figure 3 shows images of the crystals obtained for a wide range of applied endcap voltages (which set the value of *ω*_*z*_). Comparing each image with simulations, it is possible to arrive at value of *ω*_2_ for each value of *ω*_*z*_. Combining equation 3 with the definition of *ω*_1_ gives


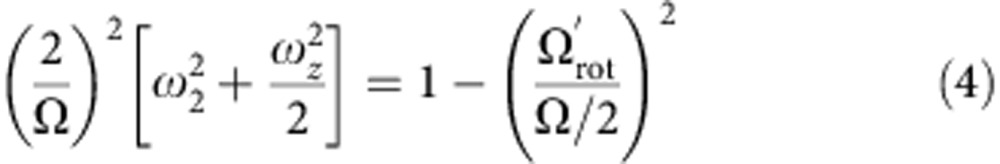


which is the equation of a circle of radius 
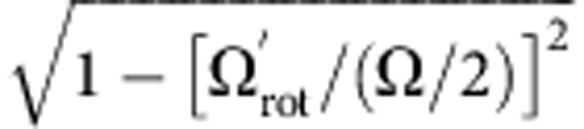
. Figure 5 is a plot of *ω*_2_/(Ω/2) versus 
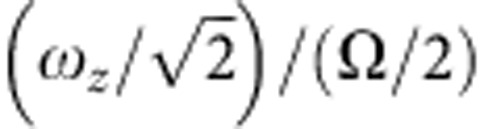
 for all the 15-ion crystal images obtained over the full range of applied voltages. For weak axial confinement, the rotation frequency of the crystal in the laboratory frame is ~Ω/4. However, when 
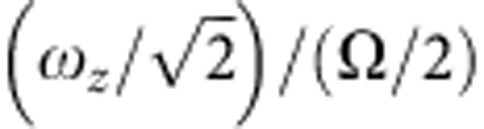
≳0.4, it can be seen that it increases. At this point, the crystal radius is ~20 μm.

As Ω_rot_ changes, the density of the crystal should adjust according to equation 1. Estimates of the density of the crystal are consistent with the rotation frequency derived by comparison of images of crystals with simulations. For a crystal having a radius less than the laser beam waist, the equilibrium rotation rate is determined mainly by the laser parameters (saturation, offset and detuning) and is approximately independent of crystal shape and size.

To achieve a high value of Ω_rot_, it is necessary to have a steep gradient of the laser beam intensity across the crystal^30^,^35^. We find that the onset of the zigzag structure of the 15-ion axial crystal is consistent with a crystal rotation frequency of ~180 kHz (0.5 × Ω/2). Further increase of the axial confinement leads to a shorter axial extent and a larger diameter of the crystal. As the radius increases, the crystal extends beyond the centre of the laser beam and comparison with simulations indicates that the rotation frequency of the crystal in the laboratory frame increases.

**Figure 6:**
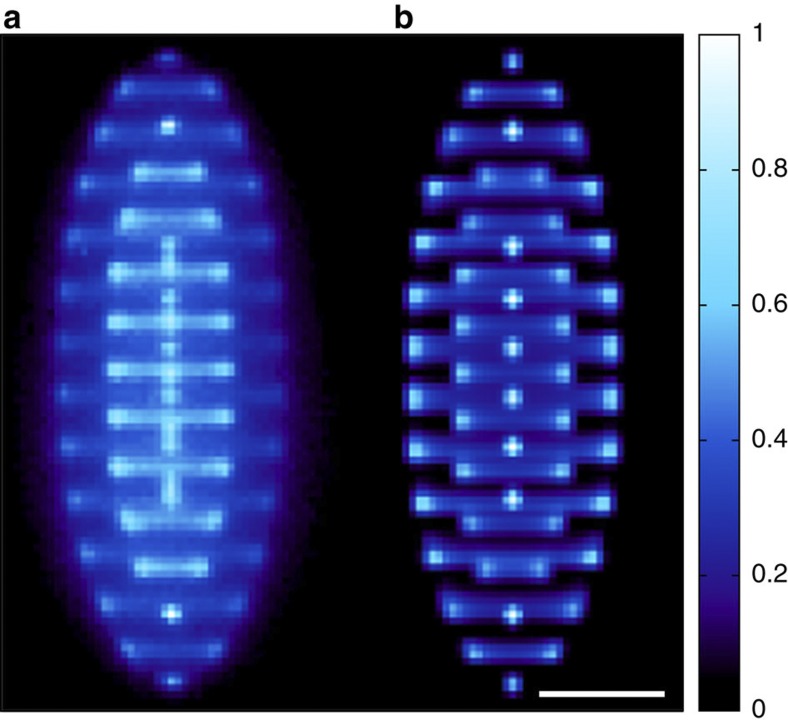
Example of a larger 3D ion Coulomb crystal. Comparison between an experimental image (**a**) of a relatively large 3D crystal and a simulation (**b**). The radial extent of the simulated crystal depends upon the rotation frequency. Optimizing the match with the experimental image allows the rotation frequency to be estimated. The best match is found for 174±4 ions with 
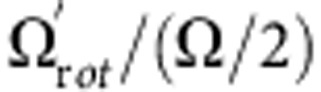
=0.66±0.01. Scale bar, 50 μm.

Figure 6 shows a larger ICC in our trap, where a number of stationary ions on the axis are surrounded by a cage of rotating rings of ions. The simulation that best fits the experimental image indicates Ω_rot_/(Ω/2)=0.34±0.01 with 
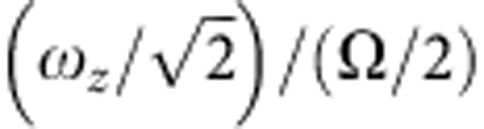
=0.28±0.01 for this crystal and that it contains 174±4 ions. However, the match is not perfect, because the conformation of the crystal may change during the exposure. For large crystals, the conformation is sensitive to any changes in the laser parameters and to perturbations from, for example, background gas collisions.

## Discussion

We have shown in this paper for the first time that it is possible to create and manipulate small Coulomb crystals of more than just two ions in a Penning trap. This is achieved without the addition of any additional oscillating electric fields as have been used in a number of other Penning trap experiments. The adaptation of our trap to provide direct axial cooling and imaging in the radial plane was the key development that allowed us to perform this work. The system has potential application in quantum simulation studies with trapped ions and is a rich system for the investigation of Coulomb crystals without micromotion.

## Methods

### Penning trap

The motion of a single ion in a Penning trap consists of an harmonic axial oscillation at *ω*_*z*_ and a superposition of two circular motions in the radial plane, the modified cyclotron motion at *ω*_+_ and the magnetron motion at *ω*_−_. These motional frequencies are given by


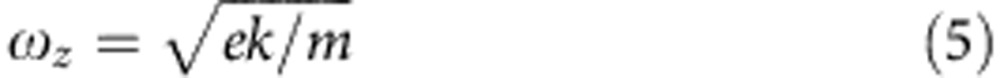



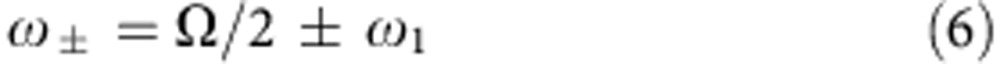


where


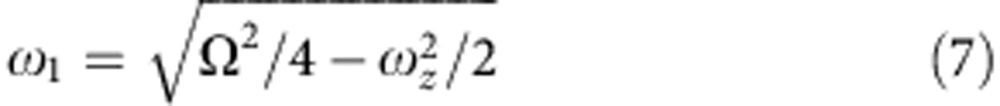


and *k*=4*U*/*R*^2^. *U* is the static voltage applied to the trap and *R* is a parameter related to the shape and size of the trap electrodes. Although Penning traps employ only static magnetic and electric fields, it is often useful to apply a weak RF drive to the electrodes of the trap. Axialization, which employs a weak oscillating radial quadrupole field at the true cyclotron frequency, has been shown to improve laser cooling of trapped ions^36^. On the other hand, the rotating wall technique that uses a rotating dipole or radial quadrupole field can be used to ‘lock’ the rotation of the ion crystal to an applied drive^28^,^27^. We have found that application of even weak oscillating potentials actually leads to excitation of motional modes of ICCs in our system so that we are able to obtain colder crystals by avoiding the use of these techniques. It is likely that this is due to imperfections in our trap, for example, misalignments or contact potentials on the electrodes.

### Deterministic loading

We load our trap by photo-ionizing neutral Ca atoms from an atomic beam *in situ* in the trap using ~5 ns pulses from a frequency-doubled YAG laser (three photons are required to reach the ionization continuum). However, loading single ions, or a well-defined number of ions, into a Penning trap is problematic. After the normal loading procedure, ions can exist in very large stable orbits in the trap (this is due to the absence of micromotion, which, in an RF trap, leads to heating of ions far from the centre of the trap). These ions may take many minutes to cool into the centre of the trap. One can therefore begin an experiment with a single ion only to find other ions later joining it at the centre of the trap, spoiling the experiment. These other ions may be either ^40^Ca^+^ ions, which are visible under laser excitation, or contaminant ions, which are not. Contaminants might be other isotopes of Ca^+^ that are not laser cooled or, for example, ionized background gas molecules. We have developed a technique that cleans the trap of contaminants by abruptly turning off the trapping potential for a short period (typically 10–20 μs) once some ions have been captured by the cooling laser and are located at the centre of the trap. Then ions that are still moving in large orbits are preferentially ejected from the trap. Our method can also be used to selectively eject ^40^Ca^+^ ions from the trap; for example, if a linear string of ions is initially loaded, turning off the trap potential briefly allows a Coulomb explosion of the ions. Over a number of seconds or even minutes, most of these ions re-cool back to the centre of the trap and rejoin the string. Once the desired number of ions has reappeared in the string, the trap voltage can be pulsed to zero again. Repeating this procedure a number of times ejects all but the required number of ions. In this way, we achieve deterministic loading of a desired number of ions.

### Cooling lasers

Zeeman splitting of the energy levels in the Penning trap leads to the requirement for multiple laser frequencies for effective laser cooling. This is achieved with two cooling lasers at 397 nm, four repumping laser frequencies at 866 nm and six repumping frequencies at 854 nm. The repumper beams are derived from single lasers at 866 and 854 nm coupled into and modulated by the same fibre electro-optic modulator (EOM). To cool all degrees of freedom efficiently, each laser beam is split into two beams, one of which is sent along the trap axis, while the other is sent into the trap in the radial plane and offset from the centre of the trap as discussed above.

### Detection system

Our current apparatus includes a considerable reworking of the optical system described in ref. 27. Principally a window has been installed at the bottom of the vacuum chamber, allowing access for a vertical (axial) laser beam with a folding mirror installed inside the vacuum chamber to steer this beam upwards. In terms of the imaging optics, for the work reported here a higher magnification was required, and as single ions and small crystals were of interest a smaller field of view could be tolerated. In the new setup, the fluorescence from the trapped ions is collected by two lens systems, each having a solid angle of 0.75% of 4*π*. One channel goes to a photomultiplier and the other to a sensitive camera (Andor EMCCD Luca R) with a magnification of 3.0±0.1. The ring electrode of the trap is divided into quadrants so that a rotating dipole field or an oscillating quadrupole field for axialization can be applied if required, although this was not used for the experiments reported here. However, the split electrode was used to compensate for patch potentials so that the DC centre of the trap was independent of the applied endcap voltage.

### Simulations

We use a simple technique to find the equilibrium configuration of an ensemble of ions in the trap. The positions of the ions are initially set at random positions and then the force on each ion, due to the confining potential and the Coulomb repulsion from all other ions, is calculated. The force from the harmonic potential along the trap axis can be directly calculated from the trap parameters. The maximum radial confinement is calculated for the frame rotating at half the cyclotron frequency. If the crystal is rotating in this frame, there is in effect an additional outward force on each ion. Moving into a frame rotating with the crystal, this is accounted for by a reduction of the confining potential in the radial plane.

After calculating the total force on all the ions in this frame, each ion is moved a small distance in the direction of its resultant force and then the calculation is repeated. The process stops after a large number of time steps when the resultant force on all ions is zero. In effect, this process simulates a highly overdamped relaxation of the ensemble to its lowest energy configuration. It is possible that the system relaxes to a local minimum of energy rather than the global minimum. To minimize this risk, the starting positions are randomized each time the simulation is performed. The results of the simulations are to be compared with experimental images of the rotating ion crystal. A simulated image is calculated by performing an angular average of the crystal structure and convolving the resulting image with the point spread function corresponding to the observed image of a single ion. By adjusting the radial potential until the simulation matches the observed image, we are able to estimate the rotation frequency of the crystal. The simulations are carried out using Matlab.

## Author contributions

R.C.T. and D.M.S. conceived the experiments. S.M., J.F.G., G.S., S.B. and D.R.C. built the Penning trap and laser cooling apparatus, and obtained preliminary data. R.T. and S.M. performed theoretical analysis. S.M. obtained the final data, wrote the simulations and carried out the data analysis. D.M.S. wrote the manuscript. All authors discussed the results and implications, and commented on the manuscript at all stages.

## Additional information

**How to cite this article:** Mavadia, S. *et al.* Control of the conformations of ion Coulomb crystals in a Penning trap. *Nat. Commun.* 4:2571 doi: 10.1038/ncomms3571 (2013).
